# Supplementary material for: IL-2K35C-moFA, a Long-Acting Engineered Cytokine with Decreased Interleukin 2 Receptor α Binding, Improved the Cellular Selectivity Profile and Antitumor Efficacy in a Mouse Tumor Model
Source: Cancers (Basel). 2022 Sep 28;14(19):4742. doi: 10.3390/cancers14194742 (PMC9563011; doi:10.3390/cancers14194742)
Supplement: Supplementary file 1 [file cancers-14-04742-s001.zip › cancers-1902643-supplementary.pdf]

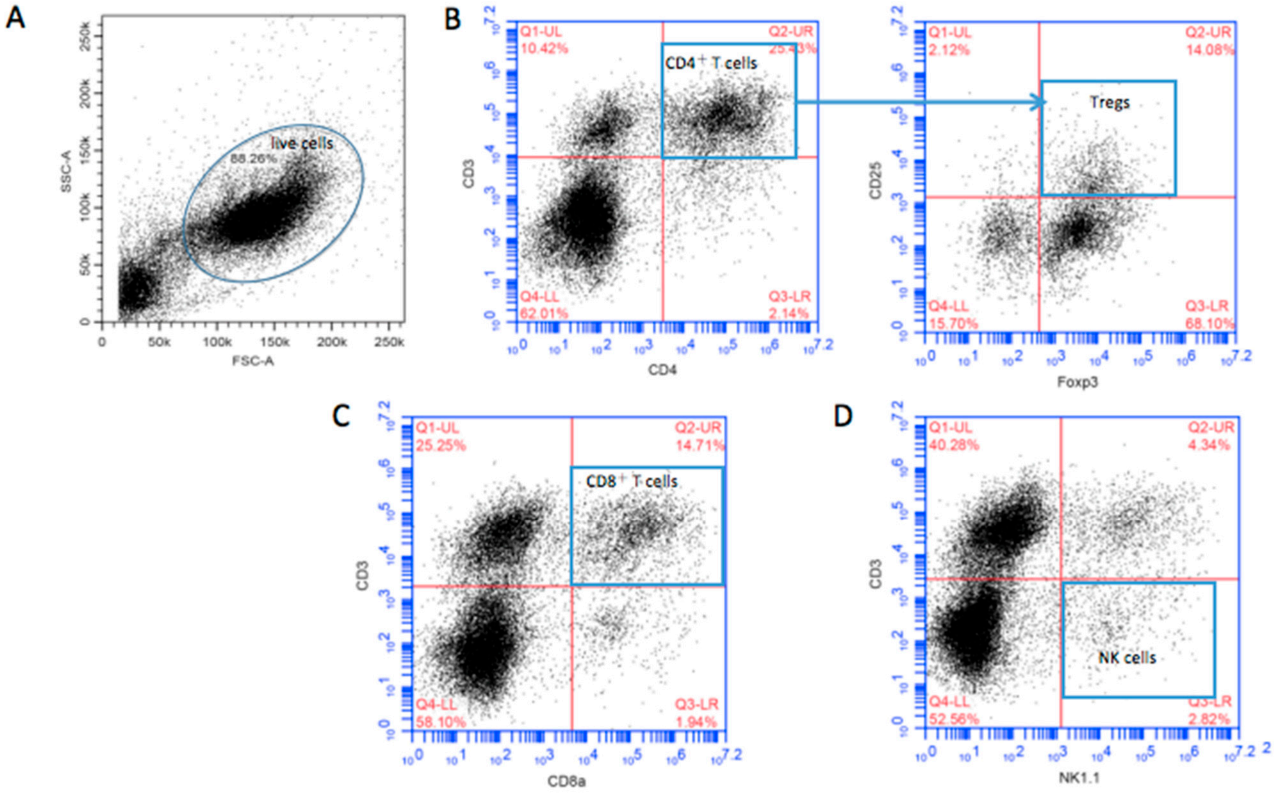

Figure S1. Representative flow gating for live cells and lymphocyte populations. (A) SSC-A vs.FSC-A gating for live cells. (B) $CD3^+$,$CD4^+$ for CD4 T cells then gated for $CD25^+$,$FoxP3^+$ Treg cells. (C) $CD3^+$, $CD8^+$ for CD8 T cells (D) $CD3^-$, NK1.1$^+$ for NK cells.

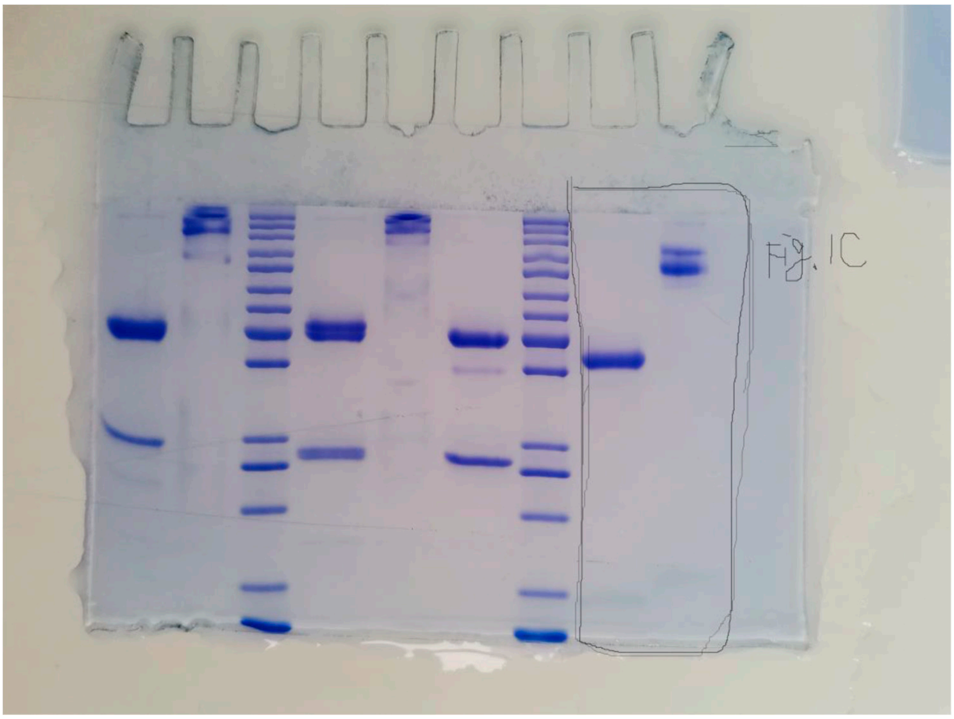

Figure S2. Original Western Blot for Figure 1C.

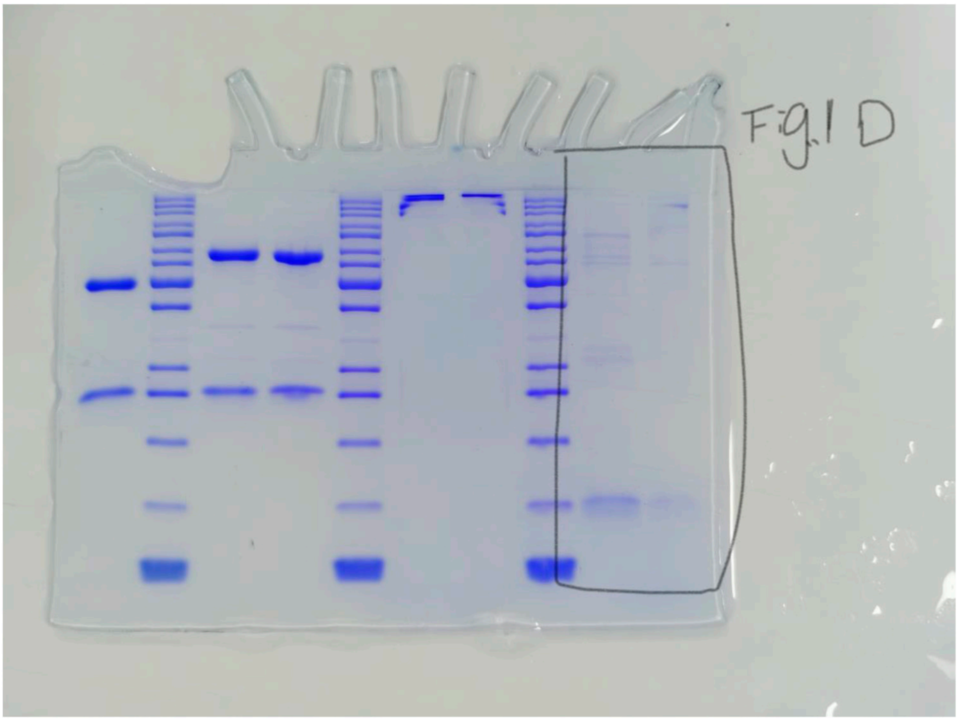

Figure S3. Original Western Blot for Figure 1D.

Table S1. Percentages of different cell types in spleens and P values.

		No.1	No.2	No.3	No.4	No.5	No.6	P value
Tregs (% live cells)	PBS	3.58	1.98	2.89	3.03	1.13	2.57	<0.0001
	IL-2WT	15.3	10.28	14.78	14.2	16.78	18.22	<0.001
	IL-2K35C-moFA	6.87	3.57	4.77	6.24	4.85	5.58	<0.0001
NK(% live cells)	PBS	2.82	3.35	2.14	5.01	4.4	2.28	<0.0001
	IL-2WT	18.26	19.38	14.26	18.4	17.81	20.75	<0.0001
	IL-2K35C-moFA	23.21	17.64	19.21	18.64	18.26	21.62	=0.2258
$CD8^+$ T(%live cells)	PBS	14.71	6.17	8.8	10.27	12.04	7.8	<0.0001
	IL-2WT	48.54	62.61	37.55	54.42	53.62	60.12	<0.0001
	IL-2K35C-moFA	58.21	37.47	40.58	49.69	58.33	24.76	=0.2476
